# Citric Acid Loaded Hydrogel-Coated Stent for Dissolving Pancreatic Duct Calculi

**DOI:** 10.3390/gels10020125

**Published:** 2024-02-03

**Authors:** Jing Li, Yanwei Lv, Zheng Chen, Jiulong Zhao, Shige Wang

**Affiliations:** 1 School of Materials and Chemistry, University of Shanghai for Science and Technology, No. 334 Jungong Road, Shanghai 200093, China; 2 Public Experiment Center, University of Shanghai for Science and Technology, No. 334 Jungong Road, Shanghai 200093, China; 3 Department of Gastroenterology, Changhai Hospital, Naval Medical University, No. 168 Changhai Road, Shanghai 200433, China

**Keywords:** pancreatic lithiasis, calculi, drug-eluting stent, hydrogel, citric acid

## Abstract

In recent years, the incidence of chronic pancreatitis has increased significantly. Pancreatic calculi obstruct the pancreatic duct and induce abdominal pain in the patients. Pancreatic duct stenting is the major treatment option for chronic pancreatitis with calculi. In this study, a new kind of drug-eluting stent, a pancreatic stent coated by methacrylated gelatin (GelMA) hydrogel loaded with citric acid (CA), was designed for the interventional treatment of pancreatic duct calculi. The CA loading capacity reached up to 0.7 g CA/g hydrogel-coated stent. The GelMA hydrogel coating has higher mechanical strength and lower swelling performance after loading with CA. The in vitro experiments of stents exhibited good performance in CA sustained release and the calculi can be dissolved in almost 3 days. The stents also showed good blood compatibility and cell compatibility. This research has important clinical value in the treatment of chronic pancreatitis with pancreatic calculi.

## 1. Introduction

Chronic pancreatitis is a progressive and irreversible chronic inflammatory disease of pancreatic tissue [1]. Pancreatic duct calculi are the characteristic pathological manifestations of chronic pancreatitis. The overall incidence of pancreatic lithiasis in the normal population is less than 1%, while about 90% of patients with chronic pancreatitis are accompanied by pancreatic lithiasis. Because of the presence of stones, the pancreatic duct is obstructed, which leads to an increase in upstream hypertension and pancreatic parenchymal tissue pressure, and even ischemia, which leads to abdominal pain [2]. Pancreatic calculi damage pancreatic internal and external secretion functions. In addition, long-term calculi irritation of the pancreatic duct can induce pancreatic cancer or secondary diabetes mellitus. Associated pancreatic exocrine insufficiency leads to malnutrition with weight loss, which severely affects patients’ work and life [3].

At present, the therapy of pancreatic duct lithiasis mainly includes extracorporeal shock wave lithotripsy, interventional endoscopic management, and medical management [4]. Over the past few years, endoscopic methods have been implemented to extract small calculi from the pancreatic duct. Small calculi can be removed using endoscopic methods such as balloon and basket sweeping [5,6]. Extracorporeal shock wave lithotripsy is a safe and effective procedure for large pancreatic calculi that are not extractable via the standard endoscopic retrograde cholangiopancreatography techniques [7]. Medical management involves the dissolution of pancreatic calculi using chemicals such as trimethadione and citrates [8,9,10]. Trimethadione is a common antiepileptic agent that causes significant damage to the liver and kidney and is converted to dimethadione by metabolic demethylation in the human liver. Dimethadione is a lipid-soluble weak organic acid that diffuses rapidly to alkaline fluid compartments and dissolves pancreatic stones by releasing protons in pancreatic juice [11]. There are few drugs and medical management can be taken into consideration for patients who have poor compliance with other treatment methods [4].

The pancreatic duct calculi consist chiefly of calcium carbonate or calcium phosphate, or even occasionally calcium oxalate [12]. The literature shows that tartaric acid and its salts, citric acid (CA) and citrate, are good chelators of calcium. The coordination chemistry between chelators and Ca^2+^ ions is the main chemical basis of these drugs to dissolve calculi in the kidney or common bile duct [13,14,15]. CA is an organic weak acid and widely exists in fruits such as lemons, oranges, and pineapples. In addition to being a good chelating agent for calcium ions, CA can also provide protons to dissolve the calculi directly in the human body. CA is also one of the main metabolites in living organisms that is formed in the tricarboxylic acid cycle or that may be introduced with diet. Currently, the use of trimethadione or citrate to dissolve pancreatic calculi in clinical studies is via oral administration, although some studies have examined the injection of the above drugs into the duodenum. However, oral drugs such as trimethadione have side effects, the surgical process of direct infusion of citrate may cause additional pain to patients, and the problem of postoperative recurrence of calculi still exists.

Chronic pancreatitis patients are often accompanied by pancreatic duct stenosis; therefore, it is necessary to implant pancreatic duct stents to dilate the pancreatic duct for safe drainage. Pancreatic duct stenting using endoscopy or surgery showed good clinical prospects for the management of benign and malignant pancreatic diseases [16,17,18]. Pancreatic duct stenting is the major treatment option for chronic pancreatitis [19,20]. However, the above therapeutic methods have some problems, such as incomplete calculi removal, calculi recurrence, and stent obstruction. Drug-eluting stents are classified as new-generation stents wrapped with a thin polymeric membrane loaded with a specific medicine. Over the past few years, drug-eluting stents have been widely used for the treatment of aneurysms, coronary heart disease, and other artery diseases [21,22]. Wan et al. fabricated a new kind of metal stent coated with drug-loaded electrospun nanofibers for dissolving biliary stones. In the study, ethylene diamine tetraacetic acid and sodium cholate were used as a litholytic for biliary stones. As a powerful chelating agent for most metal ions, EDTA was used as the dissolving agent for treating calcium bilirubinate gallstones using biliary infusion techniques. Sodium cholate is normally obtained from animal bile juices and is a kind of cholate mixture that has the potency to dissolve cholesterol stones [23]. In another study, Huang et al. engineered a bilayer swellable drug-eluting ureteric stent as a sustained drug delivery platform technology that enhances localized drug delivery to the highly impermeable urothelium, for the treatment of urothelial diseases. The bilayer coatings that are applied on a bare polyurethane ureteric stent comprise a biodegradable polymer layer loaded with a model anti-proliferative drug, mitomycin C, and a layer of hydrogel [24]. At present, endoscopic stents for chronic pancreatitis mainly include plastic pancreatic duct stents and fully coated self-expanding metal stents [25,26,27]. 

Drug-eluting pancreatic stents combine stents with calculi-dissolving drugs, of which the directed drug delivery and release can improve stone-dissolving efficiency and eliminate side effects caused by oral drugs. Moreover, the slow and sustained litholytic release of the drug-eluting stent can dissolve the calculi that cannot be taken out with endoscopic management, prevent postoperative recurrence of calculi, and alleviate pancreatic stent obstruction. The drug-eluting pancreatic stent is a good choice for pancreatic calculi and can improve the therapeutic effect of bare stent implantation in the endoscopic management of chronic pancreatitis. However, there are few studies of pancreatic calculi-dissolving drugs, and no drug-eluting stent for chronic pancreatitis with calculi have been developed and applied clinically. 

Polydopamine coating is a simple surface functionalization method inspired by mussels. Messersmith et al., in 2007, first reported a method to form multifunctional polymer coatings through simple dip-coating of objects in an aqueous solution of dopamine, in which the self-polymerization of dopamine under alkaline conditions (pH > 7.5) was used to form thin, surface-adherent polydopamine films on a wide range of inorganic and organic materials, including noble metals, oxides, polymers, semiconductors, and ceramics [28,29]. Polydopamine is well-known for its biocompatibility and excellent coating properties and has become a popular candidate for scientific research and technological innovations [30]. In this study, the coating of polydopamine was used to improve the adhesion between the hydrogel coating and the plastic pancreatic stents.

Hydrogels are three-dimensional reticulated colloidal substances that are cross-linked by the monomer or polymer side chains. As an ideal drug-delivery system, the hydrogel has an excellent loading capacity and sustainable release behavior, as well as tunable physical and chemical properties to adapt to various biomedical scenarios. Therefore, hydrogels have been widely used for tumor drug delivery, nerve repair, and transdermal administration (such as wound dressing) [31,32,33]. Among different categories of hydrogels for biomedical and tissue engineering applications, methacrylated gelatin (GelMA) hydrogel is a gelatin derivative whose precursor natural material, gelatin, is mainly a denatured collagen product [34]. GelMA hydrogel has been widely used to control drug delivery due to its good biocompatibility, biodegradability, and tunable mechanical strength [35,36]. 

In this study, a new drug-eluting stent, a pancreatic stent coated by GelMA hydrogel loaded with CA (GelMA-CA hydrogel), was designed for the potential application in the interventional treatment of pancreatic duct calculi. After modification by polydopamine, the medical plastic pancreatic stent was coated with GelMA-CA hydrogel via in situ polymerization of GelMA. It was proven that the hydrogel-coated stents have good biocompatibility. Further in vitro experiments of the hydrogel-coated stent showed good performance in CA controlled-release and dissolving of pancreatic calculi.

## 2. Results and Discussion

### 2.1. The Design and Characterization of the Drug-Eluting Pancreatic Stent

**Figure 1:**
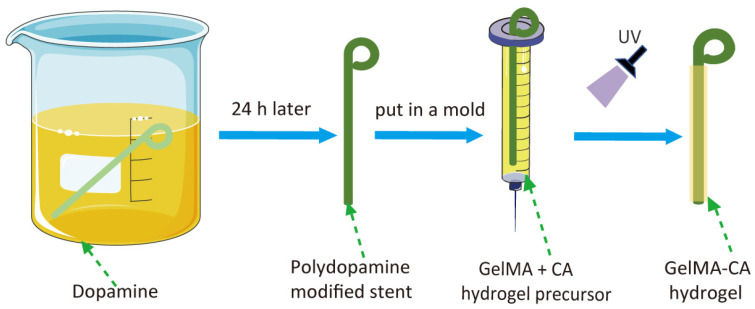
The schematic illustration of GelMA-CA hydrogel-coated pancreatic stents.

The design of GelMA-CA hydrogel-coated pancreatic stents is illustrated in Figure 1. A commercial plastic pancreatic stent was first modified with a layer of polydopamine on the surface to improve the adhesion. The polydopamine coating was applied via the self-oxidation and self-polymerization of dopamine in an alkaline solution. Then, the GelMA-CA hydrogel was formed in situ on the surface of the stents in a mold via the irradiation of UV light. 

**Figure 2:**
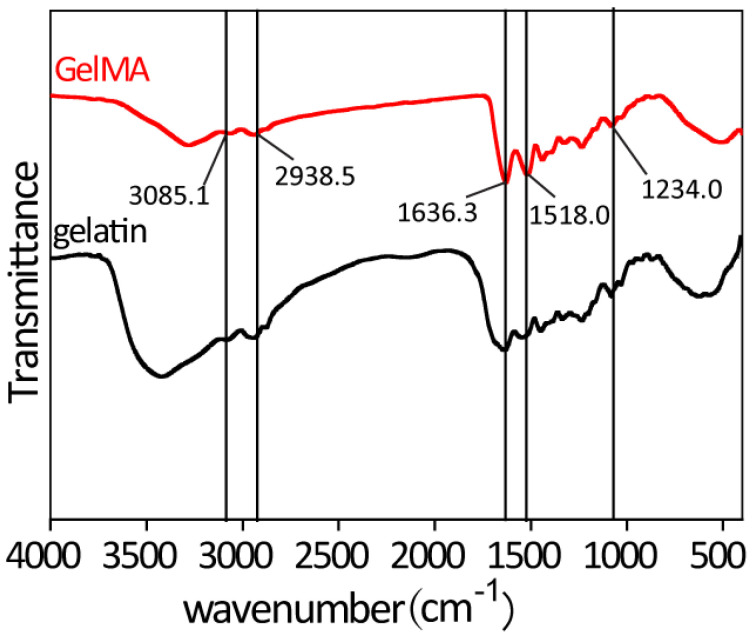
The infrared spectra of gelatin and GelMA.

The infrared spectra of gelatin and GelMA at 4000–400 cm^−1^ are shown in Figure 2. The N-H stretching vibration peak (located at 3085.1 cm^−1^), C-H stretching vibration peak (located at 2938.5 cm^−1^), C=O bending vibration peak (located at 1636.3 cm^−1^), and N-H bending vibration peak (located at 1518.0 cm^−1^ and 1234.0 cm^−1^) are the characteristic peaks of the amide structure of gelatin [37]. Compared with the infrared spectra of gelatin and GelMA, these characteristic peaks did not change, which indicates that the protein structure of gelatin was not destroyed during the reaction of methacrylic anhydride (MA) with the gelatin macromolecular backbone.

**Figure 3:**
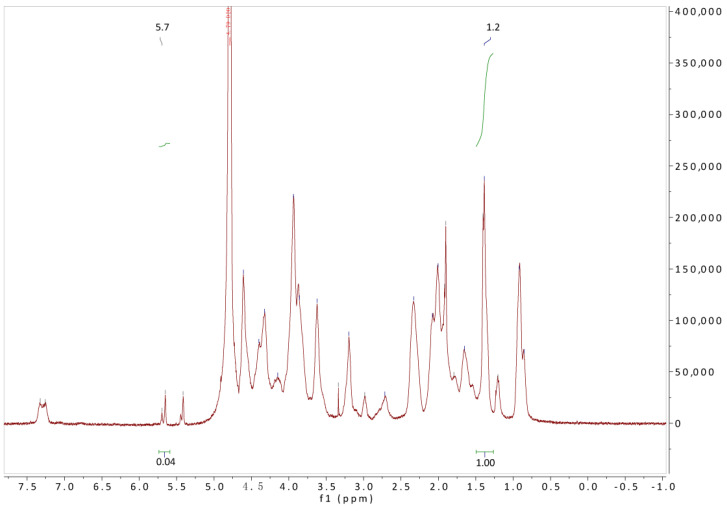
The ^1^H NMR spectra of GelMA.

The successful synthesis of GelMA was further confirmed by ^1^H NMR. In the present work, the integration at 1.2 ppm (I_1.2 ppm_) is defined as 1, and the integration at (I_5.7 ppm_) is 0.04 (Figure 3). Therefore, the methacrylation degree of gelatin was calculated as 15.4% according to Equation (1).

**Figure 4:**
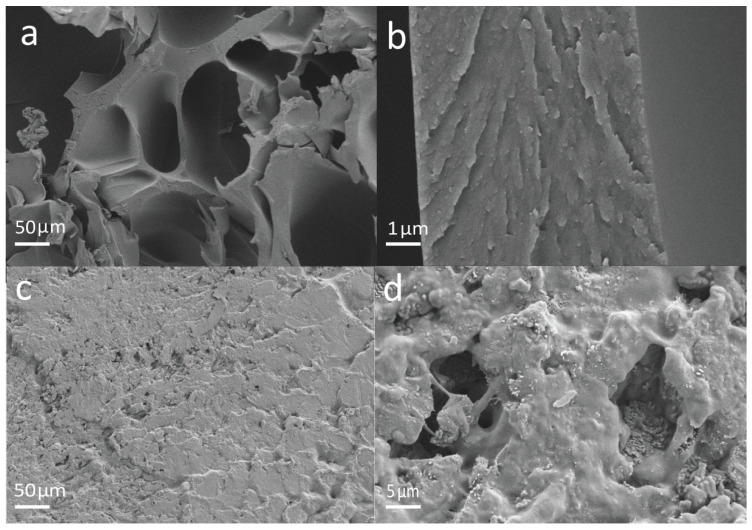
The SEM photos of (**a**,**b**) GelMA hydrogel and (**c**,**d**) GelMA-CA (CA loading content is 0.7 g/g) hydrogel (panels (**b**) and (**d**) are the local magnification of the hydrogel fracture surface in panels (**a**) and (**c**), respectively).

**Figure 5:**
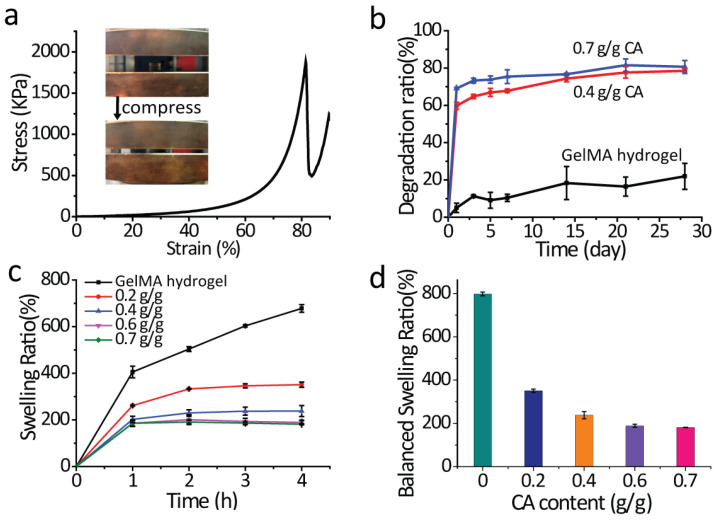
(**a**) The strain–stress curve of GelMA hydrogel; (**b**) the degradation of GelMA-CA hydrogel coating (*n* = 3); (**c**) the swelling behavior (*n* = 3); and (**d**) the balanced swelling ratio of the GelMA-CA hydrogel coating.

The SEM photos of GelMA and GelMA-CA hydrogel are shown in Figure 4. The photos show that the GelMA hydrogel has abundant pores (Figure 4a,b) and is suitable for loading CA, and the pores of GelMA hydrogel are filled with CA after loading with 0.7 g CA/g hydrogel-coated stent (0.7 g/g, Figure 4c,d). One of the major challenges of hydrogel in biomedical implants is its lower mechanical strength due to the swelling in an aqueous environment [38]. Therefore, the mechanical properties of GelMA hydrogels are one of the concerns in applications of a drug-controlled-release system. The mechanical properties of the hydrogels were characterized by compression experiments (Figure 5a). The maximum compressive stress of GelMA hydrogel was read as 1850 ± 118 kPa from the strain–stress curve, which is higher than that of other gelatin-based hydrogels in previous studies [39,40].

### 2.2. Swelling and Degradation of the GelMA-CA Hydrogel Coating

**Figure 6:**
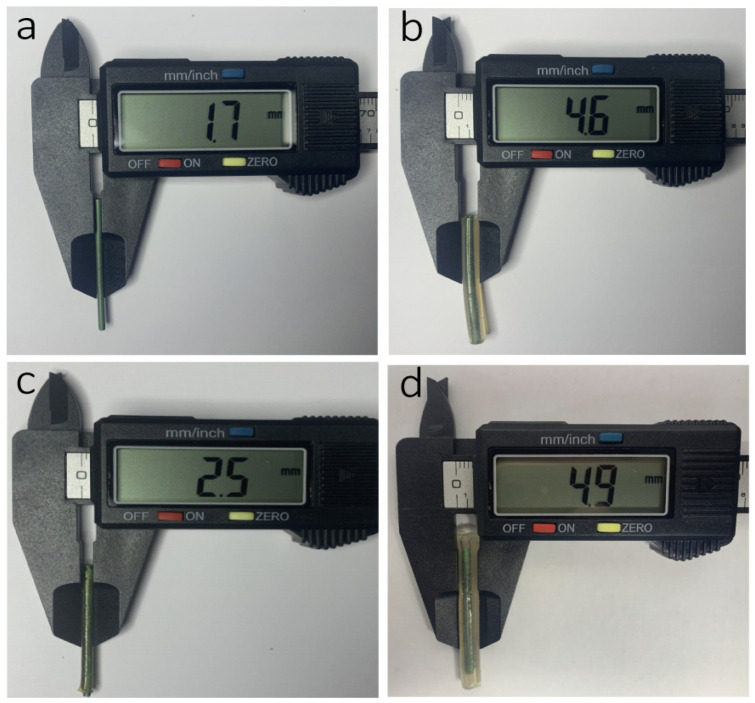
The digital photos and the diameters of the pancreatic stents at different stages ((**a**) modified with polydopamine; (**b**) coated with hydrogel; (**c**) after drying for 48 h; (**d**) after swelling for 24 h).

The swelling and degradation ability of the hydrogel is of great importance in the drug-delivery system. GelMA, acting as a carrier, can interact with the drug by covalent linking. In general, drug delivery from GelMA is mediated by diffusion and degradation [41]. At first, diffusion dominates the release profile because matrix degradation is slow [42]. Once GelMA is dissolved in the solvent, the diffusion of the drug from the porous structure occurs. Therefore, the molecular weight of drugs and the pore size of GelMA play important roles in the release process [43]. The swelling behaviors of the GelMA and GelMA-CA hydrogel in phosphate buffer solution (PBS) were studied and analyzed (Figure 5c,d). The equilibrium swelling ratio of GelMA hydrogel was 798.1% in PBS, which is similar to the swelling ratio of other GelMA hydrogels [44]. The GelMA-CA hydrogel (CA content was 0.2, 0.4, 0.6, and 0.7 g/g) reached swelling equilibrium at 4 h, and the equilibrium swelling ratio was 350.9%, 237.8%, 188.9%, and 181.5%, respectively. With the increase in CA content, the swelling ratio of GelMA-CA hydrogel decreased, which is due to the sustained release of CA and the continued absorption of water during the swelling experiment. Such a swelling ratio reduction will facilitate the application of the stent since it can alleviate the excessive surface pressure of the stent on the pancreatic duct. Figure 5b shows the degradation ratio of GelMA-CA hydrogel over time in PBS, and shows that the degradation of hydrogel is increased with the increase in CA content. The degradation ratio of GelMA-CA hydrogel (0.7 g/g) reaches 80% after 28 days. The slow degradation of hydrogel coating on the stent is also conducive to the release of CA drugs. The digital photos of the different treated stents and the diameter information of the stents are shown in Figure 6. 

### 2.3. In Vitro Release of CA and the Mechanism of the CA Release

**Figure 7:**
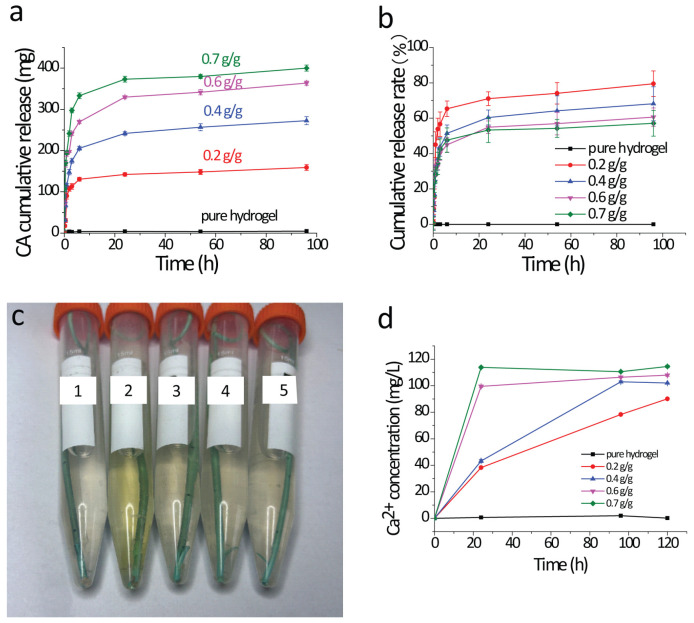
(**a**,**b**) The CA cumulative release curve (*n* = 3); (**c**) the digital photo view of the dissolution of pancreatic calculi using different GelMA-CA hydrogel-coated stents (1. pure-hydrogel-coated stent; 2. 0.2 g/g stent; 3. 0.4 g/g stent; 4. 0.6 g/g stent; 5. 0.7 g/g stent; *n* = 3); (**d**) the real-time Ca^2+^ concentration in the tubes (*n* = 3).

The CA release behaviors of the GelMA-CA hydrogel-coated stents were studied and the CA cumulative release curve is shown in Figure 7a,b. The kinetic parameters of the CA release obtained via fitting with OriginPro 8.5 (n: the release exponents, k: rate constant) are shown in Table 1.

The release of water-soluble drugs from initially dehydrated hydrogel matrices generally involves the simultaneous absorption of water and desorption of the drug via a swelling-controlled diffusion mechanism [45]. If diffusion through the material occurs faster than relaxation of the polymer chains, the kinetics is afforded by swelling and is controlled by diffusion [46]. As a water-soluble drug, the release behavior of CA by GelMA-CA hydrogel-coated stents is closely related to its swelling behavior (Figure 7a). Based on the Ritger–Peppas equation, the drug release is treated as the Fickian diffusion with n < 0.45, while the drug release is affected by the combined effects of anomalous diffusion of non-Fickian diffusion when 0.45 < n < 0.89 [47]. As seen in Table 1, the release exponents n of all the stents are below 0.45, which indicates that the release of CA follows the Fickian diffusion mechanism. At the initial rapid release stage (i.e., the first 6 h) of CA, the hydrogel absorbs water rapidly through its pores and swells rapidly (Figure 5c). The CA cumulative release rate is nearly 55–81% in the different hydrogel-coated stents (Figure 7b). The CA cumulative release rate is decreased with the increase in the CA loading content, which means the hydrogel-coated stent can release more CA in further experiments.

### 2.4. In Vitro Dissolution of Calculi

The in vitro dissolution of calculi of GelMA-CA hydrogel-coated stents is shown in Figure 7c,d. After incubation in the oscillating incubator at 37 °C for 3 days, the calculi in the centrifuge tube still existed when the hydrogel-coated stents were loaded without or with 0.2 g/g of CA, while the calculi had been dissolved and turned into flocculent when the stents were loaded with 0.4 g/g, 0.6 g/g, and 0.7 g/g of CA. Combined with the determination of real-time Ca^2+^ content (analyzed by ICP), it can be determined that the calculi were dissolved with the GelMA-CA hydrogel-coated stents (CA loading content ranging from 0.4 to 0.7 g/g). In a previous study, the citrate concentration in pancreatic juice obtained after intraduodenal infusion of citrate can dissolve 50 mg of human pancreatic stones in vitro within 25 days [48]. Compared with this study, the calculi-dissolving effect of the GelMA-CA hydrogel-coated stent is satisfactory.

### 2.5. In Vitro Hemolysis Test and In Vitro Cytocompatibility

**Figure 8:**
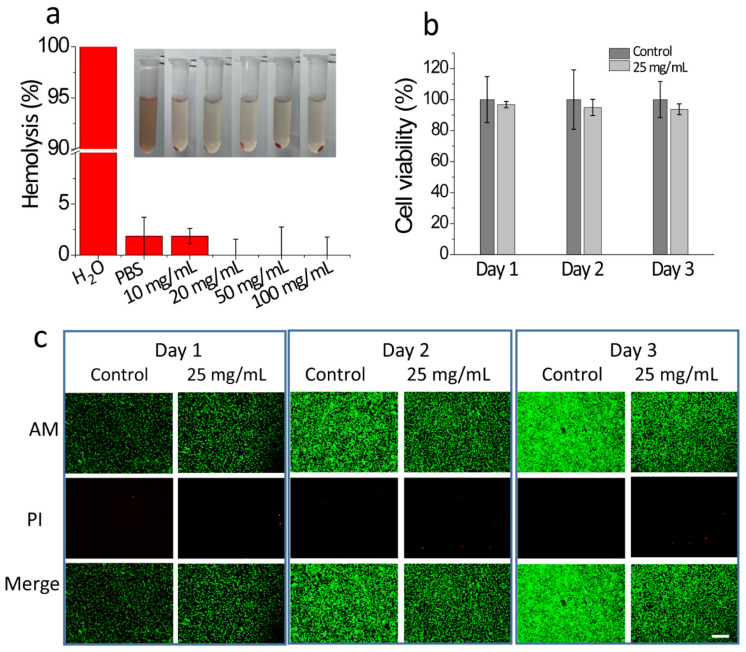
The in vitro hemolysis test and in vitro cytocompatibility of GelMA hydrogel. (**a**) The hemolysis test of GelMA hydrogel (*n* = 3); (**b**) the cell viability of GelMA hydrogel (*n* = 3); (**c**) the images of Dead/Live stained L929 cells which were cultured with the leachate of GelMA hydrogels (leach time was set as 1, 2, and 3 days, scale bar = 200 μm, corresponding to Figure 8b).

The hemocompatibility and cytocompatibility of the hydrogel-coated stents for pancreatic lithiasis are critical issues to be considered before conducting biomedical application studies. The major limitation of any blood-contacting implants and peripheral medical devices remains the blood hemolysis induced by the biomaterial. As seen in Figure 8a, the hemolysis ratios of all the experimental groups (the hydrogel concentrations were set as 10, 20, 50, and 100 mg/mL) are below 5% (a value that can be regarded as the critical level of hemolysis), which demonstrated the hemocompatibility of the hydrogel coating. The cytotoxicity of the hydrogel coating of the stents was evaluated with the CCK-8 assay and the Dead/Live staining of L929 cells which were cultured with the leachate of GelMA hydrogels (Figure 8b,c). The cell viability of all L929 cells is higher than 95% after 24 h of incubation with the leach liquor (when leach time was 1, 2, and 3 days) of 25 mg/mL GelMA hydrogel, indicating that GelMA hydrogel has good cytocompatibility and low cytotoxicity.

## 3. Conclusions

Based on the current social demand for new functional pancreatic stents, in this study, a new type of drug-eluting stent for pancreatic lithiasis was prepared via in situ synthesis of GelMA hydrogel loaded with CA on the surface of a plastic pancreatic stent. In vitro experiments demonstrated good performance of the GelMA-CA hydrogel-coated pancreatic stents in CA controlled release and pancreatic calculi dissolution. In the calculi-dissolving experiments, GelMA-CA hydrogel-coated pancreatic stents (CA contents were 0.4, 0.6, and 0.7 g/g) could completely dissolve the pancreatic duct calculi after 3 days. The prepared hydrogel-coated stent has good blood compatibility and cytocompatibility. The main drawback of this study is that the effect of the new drug sustained-release pancreatic duct stent has not been tested in vivo. To date, there are few experimental studies on in vivo pancreatic litholysis, and it is difficult to establish the animal model of in vivo pancreatic litholysis. This will be the direction of future research on drug-loaded pancreatic stents.

## 4. Materials and Methods

### 4.1. Materials

Type A gelatin (from porcine skin), Tris(hydroxymethyl)aminomethane Hydrochloride (Tris-HCl), citric acid monohydrate, Iron(III) nitrate nonahydrate, nitric acid, sodium hydroxide, and sodium chloride were purchased from Sinopharm Chemical Reagent Co. Ltd. (Shanghai, China). PBS, MA, dopamine hydrochloride, and 2-Hydroxy-4′-(2-hydroxyethoxy)-2-methylpropiophenone (photoinitiator I2959) were supplied by Aladdin Scientific Corp. (Shanghai, China). The pancreatic stents (SPSOF-5-10, batch number C1783145) were obtained from Cook Ireland Limited (Limerick, Ireland). The pancreatic duct calculi were supplied by Changhai Hospital, Naval Military Medical University. The Cell Counting Kit (CCK-8, CK04, Lot. VG533) was obtained from Dojindo Laboratories (Kumamoto, Japan). The Calcein/PI Live/Dead Viability/Cytotoxicity Assay Kit (C2015M, Lot. 121522230328) was purchased from Beyotime Biotech. Inc. (Shanghai, China).

### 4.2. Surface Modification of Pancreatic Stents

The implementation steps of the surface modification of pancreatic stents are as follows. Firstly, the plastic pancreatic stents were ultrasonically cleaned with anhydrous ethanol and deionized water (DI water) for 15 min. Then, the pancreatic stents were placed in a Tris-HCl buffer solution (pH was adjusted to 8.5). After adding dopamine (final concentration 2 mg/mL), the solution was stirred at room temperature for 24 h. After washing with DI water several times to remove the unpolymerized dopamine on the surface and drying, the polydopamine-modified pancreatic stent was obtained.

### 4.3. In-Situ Synthesis of GelMA-CA Hydrogel on the Surface of Pancreatic Stents

Preparation of GelMA: The gelatin was dissolved in DI water (0.1 g/mL) and stirred magnetically in a water bath at 50 °C to obtain a homogeneous solution. MA (MA:gelatin = 1:10) was then added at the rate of 0.5 mL/min with a peristaltic pump. After stirring at 50 °C for 4 h, the obtained GelMA solution was purified using a dialysis bag with a cutoff molecular weight of 14 kDa (Viskase, Lombard, IL, USA) at 50 °C in DI water for 4 days to remove unreacted MA and other byproducts. The GelMA was lyophilized after dialysis for further use.

In situ synthesis of GelMA-CA hydrogel on the surface of the pancreatic stents: The modified pancreatic stent was placed in GelMA solution (0.2 g/mL) and photoinitiator I2959 (0.01 g/mL) was added. After GelMA and photoinitiator I2959 were completely dissolved, CA having a different content (0.0, 0.2, 0.4, 0.6, 0.7 g/mL) was added. GelMA-CA hydrogel was formed in situ on the pancreatic stents’ surface via irradiation with UV light (365 nm, 1.67 W/cm^2^, 1 min).

### 4.4. Characterization

GelMA was dissolved in D_2_O and the structure of GelMA was identified via ^1^H-NMR (NMR, Bruker 400 M, Fällanden, Switzerland). By comparing the ^1^H-NMR spectra, the signal peak at 1.26 ppm can be attributed to resonances in the valine, leucine, and isoleucine side chains [49]. The hydrophobic side chains can be considered chemically inert and do not participate in the reaction during the synthesis of GelMA. From the known composition (0.019 mol valine + 0.0235 mol leucine + 0.0102 mol leucine in 100 g gelatin), it can be calculated that the integral of this peak (18 protons) corresponds to 0.3162 mol/100 g [50]. In addition, the total amount of free amine groups (the proton peak at 5.7 ppm of –C=C- bond) in 100 g of type A gelatin is 0.0821 mol (0.0496 mol arginine, 0.006 mol aspartic acid, and 0.0265 mol lysine). Therefore, the methacrylation degree of gelatin was calculated using Equation (1).

(1) $$Degree\;of\;methacrylation\;\left(\%\right)\;=\;0.3162\;mol\times \frac{I_{5.7}\;ppm}{I_{1.26}\;ppm}\times \frac{100}{0.0821\;mol}$$

A Fourier transform infrared spectrometer (FTIR, Nicolet IN10, Thermo Scientific, Waltham, MA, USA) was used to determine the chemical structure of gelatin and GelMA in the wavenumber range of 4000–400 cm^−1^. The hydrogel micromorphology of GelMA-CA hydrogels was observed using scanning electron microscopy (SEM, Tescan Mira4, Brno-Kohoutovice, Czech Republic). The mechanical properties of these hydrogels were examined using a universal material tester (Zwick Roell Z2.5 TH, 2.5 kN, Ulm, Germany). The GelMA-CA hydrogel was prepared as a cylinder with a diameter of 10 mm and a height of 4 mm. To test the compression resistance of the hydrogel, the sensor compressed against the hydrogel at 30 mm/min and the change in hydrogel stress was observed for a strain of 0–90%. The maximum compressive strength of the hydrogel was read from the stress–strain curve (*n* = 3).

### 4.5. Swelling and Degradation of the GelMA-CA Hydrogel

To investigate the swelling behaviors of the GelMA-CA hydrogel in PBS, the lyophilized GelMA-CA hydrogels (CA content was 0.0, 0.1, 0.2, 0.4, 0.6, and 0.7 g/g, *n* = 3) were weighted (recorded as W_0_) and immersed in PBS (0.2 mol/L, pH = 7.4). The hydrogel was removed after incubation at 37 °C for 1 h, 2 h, 3 h, 4 h, 6 h, 12 h, and 24 h, and gently drained with filter paper and weighed (recorded as W_t_). The swelling performance of hydrogel was calculated as shown in Equation (2), and the swelling kinetics curve was drawn.

(2) $$Swelling\;ratio=\frac{W_{t}-W_{0}}{W_{0}}\;\times \;100\%$$

To investigate the degradation of the GelMA-CA hydrogel in PBS, the lyophilized GelMA-CA hydrogels (CA content was 0.0, 0.4, and 0.7 g/g, *n* = 3) were weighed (recorded as W_0_) and immersed in PBS (0.2 mol/L, pH = 7.4). The hydrogel was taken out after incubation at 37 °C for 1 day, 3 days, 5 days, 7 days, 14 days, 21 days, or 28 days, and lyophilized for weighing (recorded as W_t_). The degradation ratio of the GelMA-CA hydrogel was calculated with Equation (3).

(3) $$Degradation\;ratio=\frac{W_{0}\;-\;W_{t}}{W_{0}}\;\times \;100\%$$

### 4.6. In Vitro Release of CA

The prepared GelMA-CA hydrogel-coated pancreatic stents (CA content was 0.0, 0.1, 0.2, 0.4, 0.6, and 0.7 g/g, *n* = 3) were immersed in 10 mL of PBS in 15 mL centrifuge tube and incubated in an oscillating incubator at a rate of 90 rpm. A 1 mL sample was collected at predetermined time intervals (5 min, 15 min, 30 min, 45 min, 1 h, 2 h, 3 h, 6 h, 24 h, 54 h, and 96 h) and 1 mL of PBS was added every time. The samples were placed in 25 mL colorimetric tubes, and Fe(NO_3_)_3_ (4.0 mL 0.1 mol/L) and HNO_3_ (1.0 mL 0.1 mol/L) were added. After diluting to 25 mL with DI water, the absorbance at 490 nm was measured using a visible spectrophotometer (722, Jinghua, Shanghai, China). The CA content in the sample was calculated according to the standard curve of CA. In order to study the release mechanism of CA from the GelMA-CA hydrogel-coated stents, the CA cumulative release data of different stents were modeled based on the Ritger–Peppas equation [51] with OriginPro 8.5 software. The nonlinear curve fitting equation was first defined as Equation (4). M_t_ is the drug released at time t, M_∞_ is the final released amount, k is the constant incorporating the structural characteristics of the drug-eluting stents, and n is the release kinetics exponent used to identify the mechanism types of drug release in the matrix [47].

(4) $$\frac{M_{t}}{M_{\infty }}=kt^{n}$$

### 4.7. In Vitro Dissolution of Calculi

The prepared GelMA-CA hydrogel-coated pancreatic stents (CA content was 0.0, 0.2, 0.4, 0.6, and 0.7 g/g, *n* = 3) and the calculi (with similar size and weight) were immersed in 10 mL of DI water in 15 mL centrifuge tubes and incubated in an oscillating incubator at a rate of 90 rpm. A quantity of 1 mL of sample was collected according to the predetermined time intervals and 1 mL of DI water was added, and the calculi were considered to be completely dissolved when they broke into at least 3 pieces. The Ca^2+^ concentrations in the samples were measured via ICP-MS (Avio 200, PerkinElmer, Waltham, MA, USA).

### 4.8. In Vitro Hemolysis Test

The blood compatibility of the hydrogel was assessed via the in vitro hemolysis test using the blood of KM mice (provided by Changhai Hospital, Naval Medical University). The blood was centrifuged (5000 rpm, 5 min) and rinsed three times with PBS. The collected erythrocytes were dispersed in PBS (2% *v*/*v*) for further use. The above erythrocyte suspension (0.6 mL) incubated with PBS (2.4 mL) was defined as the negative control, and the erythrocyte suspension (0.6 mL) incubated with DI water (2.4 mL) was defined as positive control. For the experimental groups, the erythrocyte suspensions (0.6 mL) were incubated with different concentrations of hydrogel coatings of the stents (10, 20, 50, and 100 mg/mL, *n* = 3). The above-treated erythrocyte suspensions were incubated at 37 °C for 2 h and then centrifuged (3000 rpm, 5 min). The absorbance of the supernatant was measured at 541 nm with an ultraviolet-visible spectrophotometer (UV1800PC, Jinghua, Shanghai, China). The absorbance of the supernatant of the negative control, positive control, and experimental groups was expressed as *A_nc_*, *A_pc_*, and *A_s_*, respectively. The hemolysis ratio was calculated according to Equation (5).

(5) $$Hemolysis\;ratio=\frac{A_{s}-A_{nc}}{A_{pc}-A_{nc}}\times 100\%$$

### 4.9. In Vitro Cytocompatibility

The in vitro cytocompatibility of the GelMA hydrogel was investigated with a mouse fibroblast (L929 cells) model (*n* = 3). The lyophilized and sterilized hydrogel coatings (25 mg/mL) were incubated in Dulbecco’s Modified Eagle medium to obtain leach liquor with different extraction times (1 day, 2 days, and 3 days). L929 cells were incubated with the leach liquor for 24 h, and then the CCK-8 solution was added to the culture; after incubation for 1 h, the absorbance of the mixture was measured at 450 nm. The L929 cells were stained using a Calcein/PI Live/Dead Viability/Cytotoxicity Assay Kit, and the staining status of the L929 cells was observed under a fluorescence microscope (the green fluorescence of Calcein AM represents the live cells, and the red fluorescence of propyl iodide PI represents the dead cells).

## Figures and Tables

**Table 1:** Kinetic parameters of the CA release obtained from the Ritger–Peppas model with different GelMA-CA hydrogel-coated stents.

Parameter	0.2 g/g Stent	0.4 g/g Stent	0.6 g/g Stent	0.7 g/g Stent
k	69.72	107.23	163.13	191.43
n	0.20	0.23	0.20	0.19

## Data Availability

The data presented in this study are available on request from the corresponding author. The data are not publicly available due to ethical.
